# Pan-cancer analysis of N4-acetylcytidine adaptor THUMPD1 as a predictor for prognosis and immunotherapy

**DOI:** 10.1042/BSR20212300

**Published:** 2021-12-07

**Authors:** Kuangxun Li, Junzhe Liu, Xinyu Yang, Zewei Tu, Kai Huang, Xingen Zhu

**Affiliations:** 1 Department of Neurosurgery, The Second Affiliated Hospital of Nanchang University, Nanchang, Jiangxi 330006, P.R. China; 2 Queen Mary School, Nanchang University, Nanchang, Jiangxi 330006, P.R. China; 3 Institute of Neuroscience, Nanchang University, Nanchang, Jiangxi 330006, P.R. China

**Keywords:** immunotherapy, N4-acetylcytidine, NAT10, pan-cancer, prognosis, THUMPD1

## Abstract

**Background:** THUMPD1 is a specific RNA adaptor that assists acetylation of mRNA and production of N4-acetylcytidine (ac4C). However, it remains unclear whether THUMPD1 plays a part in tumorigenesis and therapeutic efficacy. Here, we analyzed the expression profiles and prognostic value of THUMPD1 in pan-cancer and gained insights into the correlation between THUMPD1 expression level and immunotherapy efficacy.

**Methods:** Gene expression pattern and its correlation with prognosis, immune cell infiltration in pan-cancer were obtained from Genotype-Tissue Expression (GTEx), Cancer Cell Line Encyclopedia (CCLE) and The Cancer Genome Atlas (TCGA) databases, with Kaplan–Meier method and Spearman correlation analysis used. Western blotting and immunofluorescence on clinical samples were performed to validate our database-derived results. Correlation between THUMPD1 expression level and immunotherapy responses was also explored, based on clinical cohorts receiving programmed cell death protein 1 ligand (PD-L1) antibody therapy. Finally, gene set enrichment analysis (GSEA) was performed to show the possible tumorigenic mechanism.

**Results:** THUMPD1 was highly expressed in most cancer types, and this elevated expression indicated poor or improved prognosis for different cancers. In kidney renal clear cell carcinoma (KIRC) and rectum adenocarcinoma (READ), patients with higher THUMPD1 expression exhibited a better prognosis, while liver hepatocellular carcinoma (LIHC) patients had worse prognosis. Besides, THUMPD1 was significantly associated with immune cell infiltration, tumor mutational burden (TMB), microsatellite instability (MSI), immune checkpoints and neoantigen in many cancer types. Further, more clinical advantages and therapeutic responses were observed in patients with high THUMPD1 expression.

**Conclusions:** THUMPD1 may serve as a novel predictor to evaluate cancer prognosis and immune therapy efficacy in diverse cancer types.

## Introduction

RNA is a biologically significant macromolecule with its chemical modification highly specific and efficient to regulate RNA structures and biological functions [1]. Up to date, more than 100 natural RNA modifications of different types have been discovered, however, most of which are relatively rare in mRNA [2,3]. Previous studies had demonstrated that mRNA modification is capable to affect mRNA stability, processing and translation in post-transcriptional stage, indicating an indispensable role of mRNA modification [4,5]. Focusing on mRNA, some 11-base modifications have already been detected, one of which is N4-acetylcytidine (ac4C), the conservative chemically modified nucleoside in eukaryotic cells [2,6,7].

Earlier research suggested that ac4C is mainly present in tRNA and 18S rRNA, however, recent studies have demonstrated the existence of ac4C in mRNA as well [8,9]. Moreover, the abundance of such RNA acetylation within mRNA is even higher than the common 5′,7-methylguanosine cap [10]. Therefore, subsequent transcriptome-wide analyses were conducted to investigate the biological function of ac4C in mRNA. In 2018, Oberdoerffer et al. [5] had described a regulatory function of ac4C in mRNA expression through enhancing mRNA stability and thus improving the translation efficiency, indicating a vital role of ac4C. In most cases, RNA modification enzymes work as the holoenzyme composed of a catalytic subunit and a regulatory RNA binding subunit [11]. To produce ac4C within mRNA in human cell, N-acetyltransferase 10 (NAT10) harboring an RNA acetyltransferase domain and THUMPD1 carrying an RNA binding motif are both required [12]. Functioning as a specific ac4C adaptor, THUMPD1 is responsible to assist the catalyzation of RNA acetylation by NAT10, then ac4C is produced [13]. Furthermore, previous studies had demonstrated a significant increase in modified ribonucleotides (including ac4C) in urine of patients with several cancer types [14–19]. THUMPD1 expression test was also conducted, and showed the increased expression in breast cancer tissue compared with normal breast tissue [20]. These findings may indicate that ac4C is a possible biomarker of cancer. To further investigate the biological function of ac4C in cancers, previous studies of NAT10 and THUMPD1 are informative as they are the only enzyme to catalyze ac4C modification. However, available studies on THUMPD1 are quite limited at present. Hence, filling the gap in the field of THUMPD1 is greatly required.

In the present study, we evaluated the expression of THUMPD1 and analyzed its correlation with prognosis of different types of cancer based on The Cancer Genome Atlas (TCGA) database. Laboratory evidence was obtained and used to validate and convince our database-derived results. Moreover, we explored the correlation between THUMPD1 expression and immune cell infiltration, tumor microenvironment (TME) biomarkers and immune checkpoint genes. The relationship between THUMPD1 expression level and immunotherapy efficacy was also explored. Our findings provide novel insights into the role of THUMPD1 in pan-cancer that it may affect the prognosis of several cancer types, as well as being significantly associated with tumor immune regulation. Based on these, our study suggests THUMPD1 as a novel biomarker to predict prognosis and immune therapy response in diverse cancer types. Furthermore, the present study guides a promising prospect of THUMPD1 research in the future.

## Methods

### Clinical samples collection and ethical statement

The clinical kidney renal clear cell carcinoma (KIRC) and liver hepatocellular carcinoma (LIHC) samples were obtained from inpatients of the Second Affiliated Hospital of Nanchang University between 2018 and 2021. Tumor and adjacent normal tissues were frozen by liquid nitrogen and stored in −80°C until use. The present study got approval from the Medical Ethics Committee of The Second Affiliated Hospital of Nanchang University. Sample acquisition and utilization were performed according to the approved guidelines. Informed consent was obtained from each patient.

### Cell culture and immunofluorescence microscopy

U87 and LN229 glioma cells growing on coverslips were used. The cells were fixed in 4% paraformaldehyde for 1 h and incubated in 0.3% Triton X-100 for 15 min in phosphate-buffered saline (PBS) environment. Cells were then blocked in 5% goat serum for 1 h after PBS washing. Then cells were incubated with THUMPD1 rabbit polyclonal antibody (1:50, 14921-1-AP, Proteintech®) at 4ºC overnight. Following the washing step, secondary antibody Alexa Fluor 488-conjugated Goat Anti-Rabbit IgG (1:200, ab150077, Abcam, Cambridge, U.K.) was used for 1 h. Next, cells were stained by DAPI (C0065, Solarbio, Beijing, China) for 30 s, away from dark. Finally, cells were washed by PBS for three times and visualized under the fluorescence microscope (Nikon, Tokyo, Japan).

### Western blotting

Protein was extracted from clinical samples using RIPA (Applygen, Beijing, China). The prepared protein samples were separated in 10% sodium dodecyl sulfate (SDS) polyacrylamide gel at constant voltage of 90 V for 30 min and then 120 V for 60 min. Protein bands on gel were then transferred to PVDF membrane at constant current of 350 mA for 1 h. The membranes were subjected to blocking buffer containing 5% skim milk in Tris-buffered saline with 0.1% Tween 20 (TBST) at room temperature for 2 h. After the rinse, membranes were incubated with corresponding primary antibodies at 4ºC overnight. After another three-time rinse, the membrane was incubated with horseradish peroxidase (HRP)-conjugated secondary antibodies at room temperature for 2 h. Visualization of protein bands on membrane was achieved by hypersensitive enhanced chemiluminescence (ECL) kit (Thermo Fisher Scientific, Waltham, MA, U.S.A.) in GV6000M (GelView 6000pro, Guangzhou Biolight Biotechnology Co., Ltd., Guangzhou, China). Relative expression level of target proteins was normalized using GAPDH as internal control. The information of all the antibodies is provided in Table 1.

**Table 1:** List of antibody information

Antibody	Company	Host	Dilution
**THUMPD1**	Proteintech	Rabbit	1:500
**GAPDH**	Proteintech	Mouse	1:5000
**Goat anti-rabbit**	Proteintech	Goat	1:20000
**Goat anti-mouse**	Proteintech	Goat	1:20000

Proteintech: Wuhan, Hunan, China. Antibodies to be stored at −20°C.

### Data mining and processing

Expression profiles of *THUMPD1* gene in normal and cancerous tissues were acquired from three public databases: Genotype-Tissue Expression (GTEx), Cancer Cell Line Encyclopedia (CCLE) and TCGA. THUMPD1 mRNA expression data in normal tissues were obtained from GTEx, a dataset containing expression data of 31 healthy tissues, and the expression distribution in different cancer cell lines was obtained from CCLE, a database containing information of more than 1100 cancer cell lines. Differential expression data between cancerous and para-cancerous normal tissues were obtained from TCGA, a useful dataset that collects clinical data of 33 cancer types. Available information from GTEx and TCGA was downloaded from UCSC Xena platform. Genetic and epigenetic alteration data of THUMPD1 gene in pan-cancer were obtained from TCGA database. These data were then processed to evaluate the correlations between THUMPD1 expression and mismatch repairs (MMRs) and methyltransferases gene expression in pan-cancer.

### Cox regression analysis and Kaplan–Meier survival analysis

Univariate Cox regression analysis was conducted to assess the effect of THUMPD1 expression on patients’ overall survival (OS), disease-specific survival (DSS), disease-free interval (DFI) and progression-free interval (PFI) in TCGA pan-cancer cohorts using ‘forestplot’ R package. For cancer types showing significance, Kaplan–Meier analysis was performed. Low- and high-THUMPD1 expression groups were set up based on normalized gene expression data from respective cohorts in TCGA, with calculated *P*-value determined by *t* test. Hazard ratios (HRs) with 95% confidence intervals (CIs) and log-ranked *P*-value were calculated. Significant threshold was set when Cox *P*-value <0.05.

### Immune cell infiltration analysis

Tumor Immune Estimation Resource (TIMER) database is a comprehensive resource for systemic analysis of immune cell infiltration in diverse cancer types. TIMER was used to predict the abundance of tumor-infiltrating cells from gene expression profiles using a previously published deconvolution statistical method [21]. With the reservoir of 10897 samples across 32 cancer types from TCGA database, TIMER was exploited to evaluate the diversity of immune infiltration [22]. We downloaded the infiltration scores of six immune infiltrates (B cells, CD4^+^ T cells, CD8^+^ T cells, dendritic cells, macrophages and neutrophils) in tumor tissues from pan-cancer cohorts. Correlation between immune cell infiltration and THUMPD1 expression was then determined based on the infiltration scores. TME plays an important role in development and progression of tumors. There are classical biomarkers used to evaluate the condition of TME and help study the tumor immunology, such as tumor mutational burden (TMB), microsatellite instability (MSI) and neoantigens. Correlation between THUMPD1 expression and these TME biomarkers were investigated by Spearman’s correlation test. Forty-seven immune checkpoint (ICP) genes were selected from previous study [12], and their correlation with THUMPD1 expression was also estimated. *P*-value <0.05 was considered significant.

### Cohort validation of immunotherapy

A systemic study of immune checkpoint blockade gene expression profiles was performed. Gene expression and immunotherapeutic effect was obtained from ‘IMvigor210’ package [23]. According to the correlation between THUMPD1 expression and patients’ survival, the ‘surv-cutpoint’ function of ‘survminer’ R package was used to divide patients into high and low THUMPD1 expression cohorts, with the cut-off point exhibiting the maximum rank statistic. The Kaplan–Meier method and log-ranked test was used to determine the difference. Based on the two cohorts, difference of immunotherapeutic effect was investigated using Chi-square test. To future investigate immunotherapeutic response prediction in different cancers, we collected subtyping information of TCGA pan-cancer cohorts in previous study [24]. Then, THUMPD1 expression data were integrated with the subtyping materials and plotted into a boxed plot by ‘ggpubr’ R package.

### Gene set enrichment analysis

To explore the role of THUMPD1 expression on cancers, clinical samples were divided into high- and low-expression groups based on median gene expression value. Gene set enrichment analysis (GSEA) was performed to explore the THUMPD1 enrichment of KEGG and Hallmark pathways. An FDR < 0.05 was considered as the threshold of significance.

### Statistical analysis

Kruskal–Wallis test was performed to evaluate the expression difference among various tissues. The significance of the difference in gene expression between cancerous and para-cancerous normal tissues was determined by Wilcoxon test, with a significant threshold of *P*<0.05. HR and Cox *P*-value were assessed by Univariate Cox regression method. Kaplan–Meier method was used to estimate survival probability against time, with log-ranked *P*-value and 95% CI. The correlation between THUMPD1 expression and the targets of interest including immune cell infiltration scores, TMB, MSI, neoantigens, MMRs and methyltransferase was evaluated by Spearman’s correlation test. Statistical significance was set at *P*-value <0.05.

## Results

### THUMPD1 expression across normal and cancer tissues

**Figure 1:**
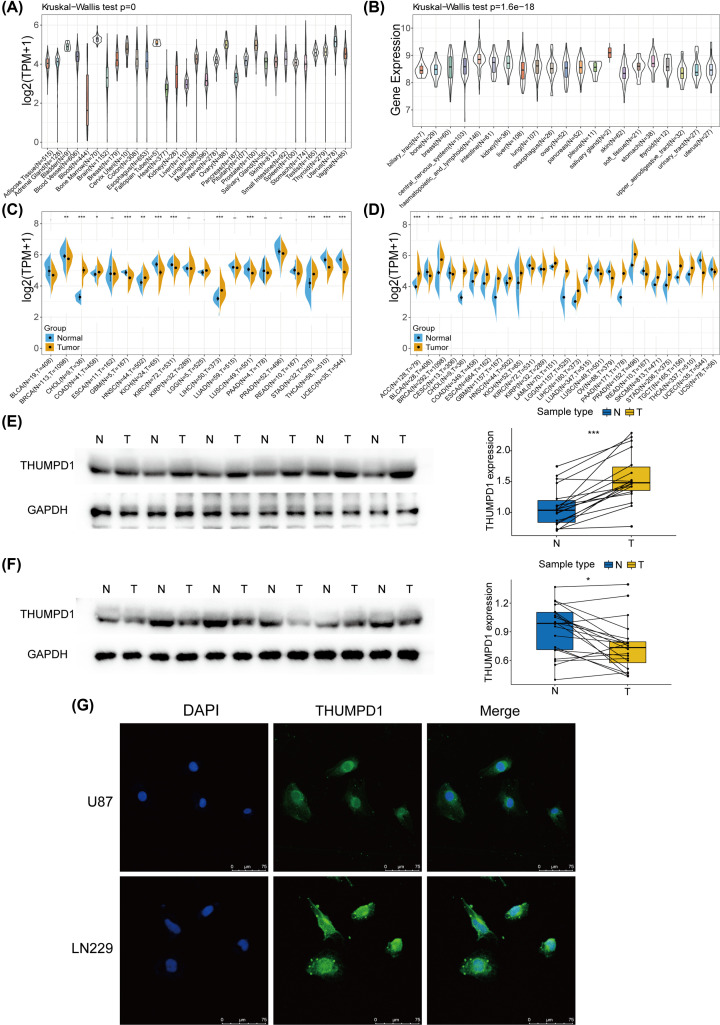
Expression level of THUMPD1 in various normal and cancer tissues (**A**) mRNA expression levels of THUMPD1 in 31 normal tissues from GTEx database. (**B**) mRNA expression level of THUMPD1 in 21 cancer tissues from CCLE database. (**C**) Differential expression of THUMPD1 mRNA between cancer and adjacent normal tissues from TCGA database. (**D**) Differential expression of THUMPD1 mRNA between cancerous and para-cancerous tissues, combining data from GTEx and TCGA databases. (**E**) Comparison of THUMPD1 protein expression between cancerous and para-cancerous normal tissues from KIRC patients. *n*=18 pairs. (**F**) Comparison of THUMPD1 protein expression between cancerous and para-cancerous normal tissues from LIHC patients. *n*=20 pairs. (**G**) Protein distribution of THUMPD1 in U87 and LN229 cell lines. Blue fluorescence, DAPI, a specific dye staining DNA to localize the cell nucleus; green fluorescence, FITC, combines with primary antibody, showing the distribution of target protein. *P<0.05, **P<0.01, ***P<0.001.

To determine the THUMPD1 expression in diverse tumor and normal tissues, mRNA data from GTEx, CCLE and TCGA databases were analyzed. According to the GTEx dataset, THUMPD1 mRNA expression was comparably expressed in different tissues, with exception of bone marrow, ovary, prostate and uterus (Figure 1A). Characterized by active differentiation, bone marrow is reasonable for its relatively higher mRNA expression level. Bladder and fallopian tube also showed a higher expression in spite of insufficient sample size (*n*=9 and 5, respectively). In CCLE database, mRNA expression data across 21 cancer cell lines were obtained and analyzed. Cancer cells had approximately two-fold increase in THUMPD1 expression compared with corresponding normal tissues (Figure 1B). Besides, the differences of expression within each group were narrower. Further comparison among all cancer cell lines suggested that salivary gland was the highest expression tissue, though its sample size (*n*=2) was quite small. In the case of enough sample size, hematopoietic and lymphoid tissues revealed the highest expression. The distribution of THUMPD1 expression in TCGA cohorts is shown in Figure 1C. Significant difference was observed in 12 out of 21 cancer types. Considering the lack of normal sample in TCGA database, information from GTEx dataset was combined with that of TCGA to analyze the expression differences among 27 cancer types. As shown in Figure 1D, our results indicated that the expression was significantly different in multiple cancers (23 out of 27). Most cancer tissues had higher THUMPD1 expression than corresponding normal tissues, such as adrenocortical carcinoma (ACC) and LIHC. Lower THUMPD1 expression was observed in bladder urothelial carcinoma (BLCA), KIRC, lung squamous cell carcinoma (LUSC), ovarian serous cystadenocarcinoma (OV) and uterine corpus endometrial carcinoma (UCEC) cohorts. The cervical and endocervical cancers (CESC), kidney renal papillary cell carcinoma (KIRP), rectum adenocarcinoma (READ) and uterine carcinosarcoma (UCS) cohorts shared similar THUMPD1 expression compared with para-cancerous normal tissues. Based on the mRNA expression pattern, we performed Western blotting to investigate the protein expression pattern of THUMPD1 in KIRC and LIHC, the representative cancer types showing opposite expression alteration in database-derived analysis (Supplementary Figure S1). As shown in Figure 1E,F, the results of THUMPD1 protein expression were consistent with its mRNA expression profiles, verifying the mRNA expression analysis above. Immunofluorescence was also performed in human cancer cells to investigate the intracellular location of THUMPD1 protein. Our result in Figure 1G demonstrated that the THUMPD1 protein was distributed in the whole cells, and the abundance in cytoplasm. Hence, our observations showed that the THUMPD1 was differently expressed in a variety of cancers, suggesting that it may play a role in cancer progression.

### Correlations between THUMPD1 expression and prognosis

**Figure 2:**
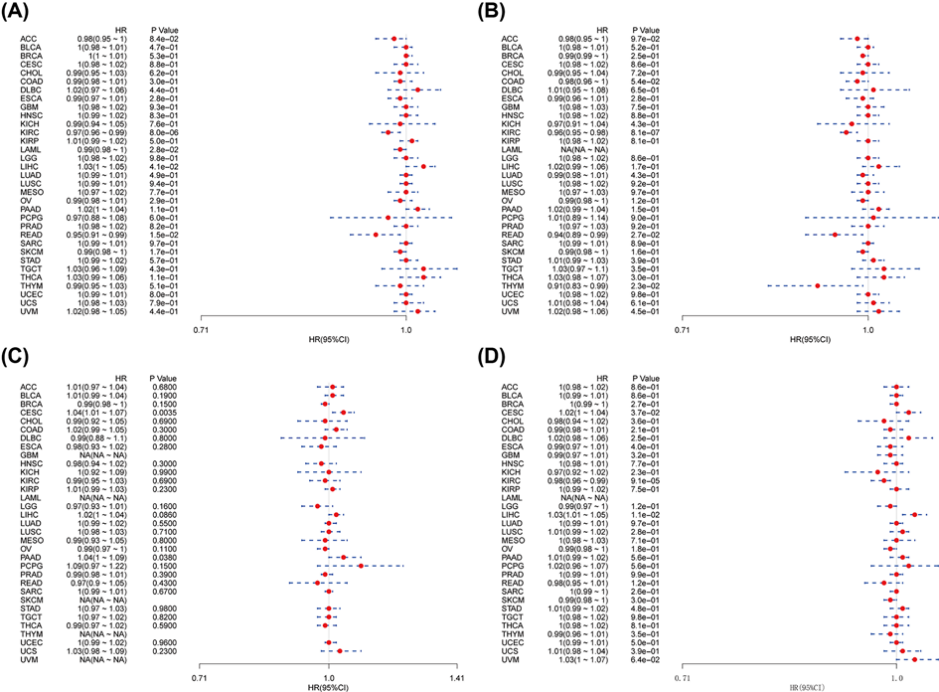
Prognosis of patients with 33 cancer types from TCGA database (**A**) Association between THUMPD1 expression and OS. (**B**) Association between THUMPD1 expression and DSS. (**C**) Association between THUMPD1 expression and DFI. (**D**) Association between THUMPD1 expression and PFI. Univariate Cox regression analysis was used, significant threshold was set at *P*<0.05.

**Figure 3:**
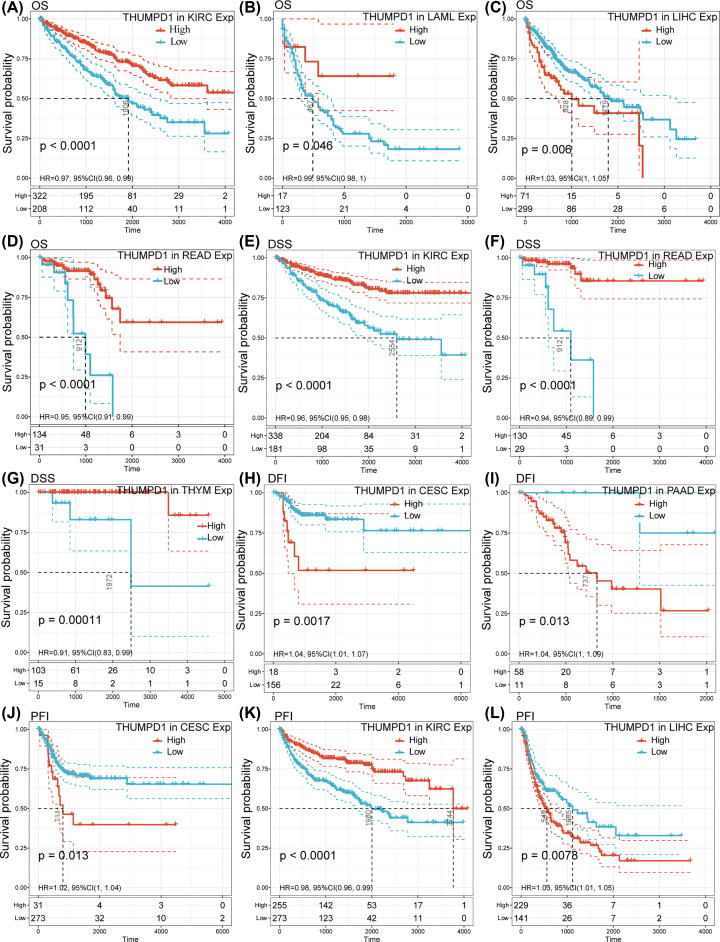
Kaplan–Meier curves comparing survival probability between high and low THUMPD1 expression cohorts (**A**–**D**) OS difference in KIRC (A), LAML (B), LIHC (C) and READ (D). (**E**–**G**) DSS difference in KIRC (E), READ (F) and THYM (G). (**H**,**I**) DFI difference in CESC (H) and PAAD (I). (**J**–**L**) PFI difference in CESC (J), KIRC (K) and LIHC (L). Grouped according to THUMPD1 mRNA expression levels in a dichotomous fashion. Abbreviations: PAAD, pancreatic adenocarcinoma; THYM, thymoma.

To evaluate the correlations between THUMPD1 expression level and OS in 33 cancer types from TCGA database, Univariate Cox regression analysis was performed. The HRs for THUMPD1 achieved significance in KIRC, acute myeloid leukemia (LAML), LIHC and READ, among which the highest protective effect was observed in READ and the highest risk effect showed in LIHC (Figure 2A). Kaplan–Meier survival analysis of these cancer types using patients’ data dichotomized for median gene expression level, showed that the survival differences were all significant, and that patients with high expression of THUMPD1 had favorable outcome in KIRC, LAML and READ, and worse outcome in LIHC (Figure 3A–D).

Considering the interference of non-cancer related deaths during follow-up period, we also investigated the relationships between THUMPD1 expression and DSS in 33 cancer types. The results of Univariate Cox regression analysis revealed a significant HR only in KIRC, READ and thymoma (THYM) (Figure 2B). Particularly, THYM had the highest protective effect (HR = 0.91). The following survival analyses of the three cancer types all suggested an improved prognosis for patients with high THYMPD1 expression (Figure 3E–G).

Further, we explored the association between THUMPD1 expression and DFI in 33 cancer types from TCGA. Significant HR showed in CESC and pancreatic adenocarcinoma (PAAD), which were both suggested to be risk factor with HR = 1.04 (Figure 2C). Following survival analyses that divided patients into high and low THUMPD1 expression indicated that earlier recurrence or metastasis after tumor resection was observed in CESC and PAAD patients with high THUMPD1 expression (Figure 3H,I).

Finally, the correlation between THUMPD1 expression and PFI in 33 cancer types was analyzed. Significant HRs were present in CESC, KIRC and LIHC (Figure 2D). In survival analysis, significant differences between high- and low-expression cohorts were observed in patients with KIRC and LIHC. KIRC patients with high THUMPD1 expression exhibited longer time to disease progression, and patients with LIHC would get progression in earlier stage (Figure 3J–L). Thus, high THUMPD1 expression improved survival probability for KIRC patients, while it shortened the time of cancer progression for CESC and LICH patients.

Overall, our results represented that THUMPD1 could function as a prognosis predictor in several kinds of cancers.

### Correlation between THUMPD1 expression and immune infiltration in pan-cancer

**Figure 4:**
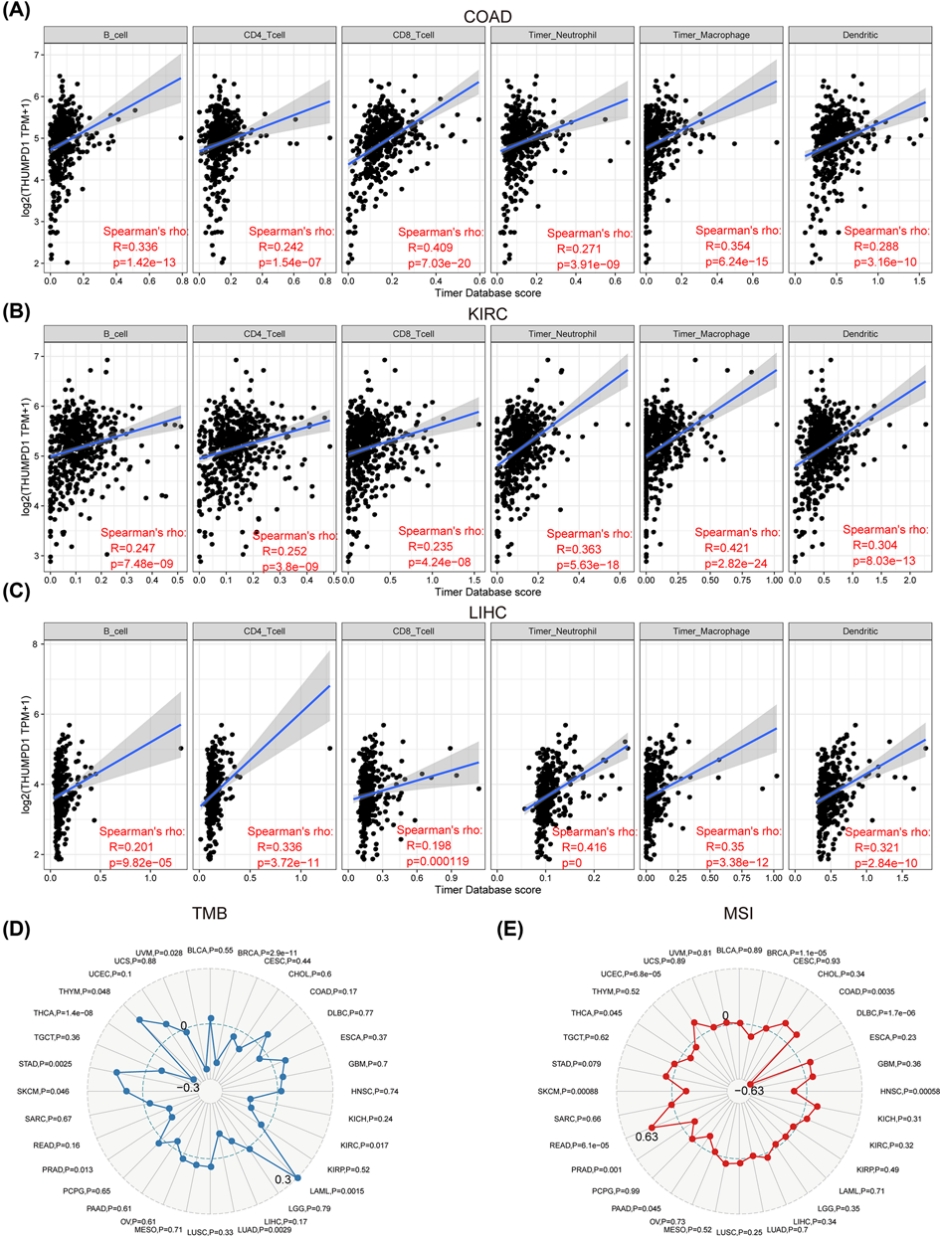
Correlation between THUMPD1 expression and tumor infiltrations (**A–C**) Correlation between THUMPD1 expression and infiltration scores of six immune infiltrates (B cells, CD4^+^ T cells, CD8^+^ T cells, dendritic cells, macrophages and neutrophils) in COAD (A), KIRC (B) and LIHC (C). (**D**) Correlation between THUMPD1 expression and TMB. (**E**) Correlation between THUMPD1 expression and MSI. The used clinical information was obtained from TCGA database. Spearman correlation test, *P*<0.05 was the significant threshold.

Tumor-infiltrating lymphocytes (TILs) are the independent predictors of sentinel lymph nodes status and survival of patients with cancer [25,26]. Hence, we investigated the possible relationships between THUMPD1 expression and immune cell infiltration in 33 cancer types from TIMER. The degree of correlation was transformed into calculated coefficient of association in each cancer type. Combining THUMPD1 expression data and infiltration scores of six immune cell types (B cells, CD4^+^ T cells, CD8^+^ T cells, dendritic cells, macrophages and neutrophils), we discovered significant correlations in several cancers, among which the top three cancers with highest infiltration scores were colon adenocarcinoma (COAD), KIRC and LIHC. The linear regression models indicated that high THUMPD1 expression might be associated with a possible increased immune infiltration level (Figure 4A–C**)**. Interestingly, among all the six immune cell types, macrophages had the maximal significant coefficient. These findings strongly suggested a vital role of THUMPD1 in immune infiltration.

### Correlations between THUMPD1 expression and TMB, MSI, neoantigen in pan-cancer

Frequent mutations in tumor cells lead to resistance against anti-tumor immunotherapy, indicating a worse prognosis of cancer patients [27]. To explore the effect of THUMPD1 on immune response in tumor tissues, we evaluated the correlations between THUMPD1 expression and TMB, MSI and neoantigen, which are reportedly prognostic biomarkers of cancer immunotherapy [28,29]. TMB is a quantifiable biomarker that helps reveal the total number of mutations in cancer cells. Hence, the correlation between TMB and THUMPD1 expression was studied by Spearman correlation analysis. The results showed significance in several cancers: breast invasive carcinoma (BRCA), KIRC, LAML, lung adenocarcinoma (LUAD), prostate adenocarcinoma (PRAD), skin cutaneous melanoma (SKCM), stomach adenocarcinoma (STAD), thyroid carcinoma (THCA), THYM and uveal melanoma (UVM), of which LAML had the highest coefficient and THCA had the lowest coefficient (Figure 4D). Among these cancer types, LAML, SKCM, STAD and THYM were positively correlated with THUMPD1 expression and the remaining were negatively associated, possibly suggesting different prognosis. As for MSI, a pattern of hypermutation in microsatellite sequence resulting from any insertion or deletion of repeated units. The association between THUMPD1 expression and MSI was evaluated using Spearman’s rank correlation analysis. Results showed the significant correlations were positive in COAD, READ and UCEC, of which READ had the highest coefficient, and the negative correlation achieved in BRCA, diffuse large B-cell lymphoma (DLBC), head and neck squamous cell carcinoma (HNSC), PAAD, PRAD, SKCM and THCA, of which DLBC had the lowest coefficient (Figure 4E). In the analyses of relationship between THUMPD1 expression and neoantigen, we found the only cancer types with significance were LUAD, BRAD and THCA, and that they were all negatively correlated (Supplementary Figure S2).

### Correlation between THUMPD1 expression and immune checkpoint genes in pan-cancer

**Figure 5:**
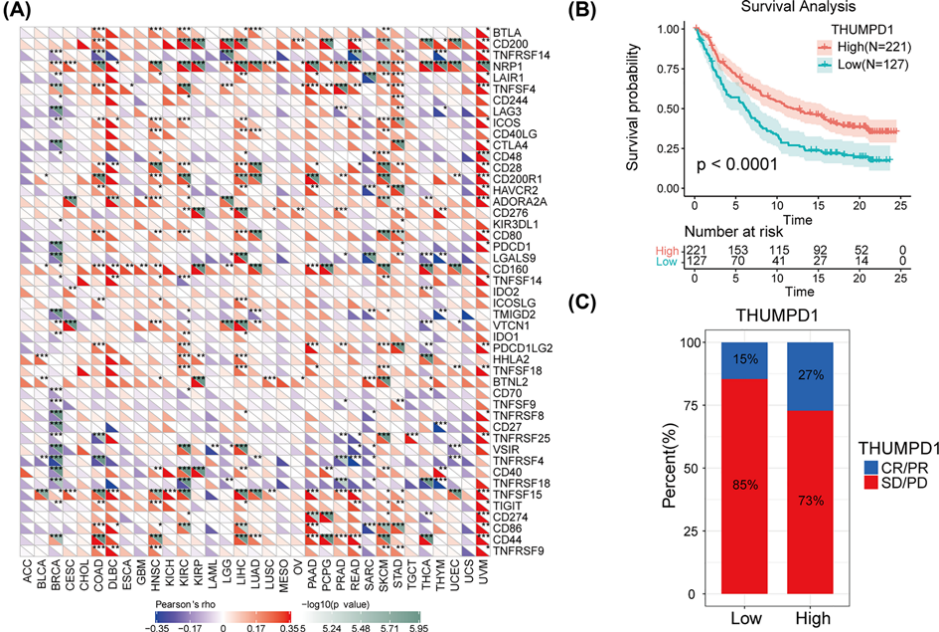
Correlation between THUMPD1 expression and immune checkpoints (ICPs) (**A**) Heatmap exhibiting the correlation between THUMPD1 and 47 ICP genes expression in 33 cancer types from TCGA database. The left bottom triangle in each unit represents coefficient of association, computed by Pearson’s correlation test; the triangle in the upper right represents the *P*-value in log10 format. *P<0.05, **P<0.01, ***P<0.001. (**B**) Survival analysis for low (*n*=127) and high (*n*=221) THUMPD1 expression groups from clinical cohorts receiving anti-PD-L1 immunotherapy. (**C**) Proportion of patients with therapeutic response to PD-L1 blockade immunotherapy in low and high THUMPD1 expression cohorts. Cut-off point was determined at the maximal statistic rank. Abbreviations: CR, complete response; PD, progressive disease; PR, partial response; SD, stable disease. Response/non-response = 15/85% in low THUMPD1 expression group and 27/73% in high THUMPD1 expression group. Abbreviation: PD-L1, programmed cell death protein 1 ligand.

Normally, tumor cells in TME are recognized and cleared by immune system. However, tumor cells evolve strategies to inhibit the function of immune system, making it fail to kill tumor cells and tumor survives in different stages of anti-tumor immune responses [30]. Immunotherapy is a novel treatment to restore normal anti-tumor immune responses by reactivating and maintaining tumor-immune cycle [12]. ICPs are the molecules on immune cells surface that modulate the degree of immune activation. Previous study had demonstrated an essential role of immune checkpoint inhibitors in immunotherapy [30]. In the present study, we explored the correlation between THUMPD1 expression and ICP genes in pan-cancer to assess the possible function of THUMPD1 in immunotherapy. Among estimated 47 immune checkpoint genes, strong significance was found in many cancer types, such as BRCA (29 of 47), COAD (20 of 47), HNSC (22 of 47), KIRC (26 of 47), LIHC (25 of 47), SKCM (27 of 47) and UVM (26 of 47). Furthermore, positive correlation between THUMPD1 expression and immune checkpoint genes was found in COAD, DLBC, HNSC, KIRC, LAML, LIHC, SKCM and UVM; negative correlation was found in ACC, BRCA, BLCA, CESC, LAML, low-grade glioma (LGG), mesothelioma (MESO), sarcoma (SARC), THCA and UCS, though, for some of the results were not to a significance (Figure 5A). Therefore, THUMPD1 may be involved in regulation of immune checkpoints, which then affect the efficacy of immune checkpoint inhibitor therapy.

### Correlation between THUMPD1 expression and immunotherapy response

Our research has demonstrated the correlation between THUMPD1 expression and immune cell infiltration, TMB and MSI, indicating an indirect correlation with immunotherapy efficacy. Here, clinical data were obtained from previously reported cohorts to assess the possible correlation between THUMPD1 expression level and immunotherapy response [23]. Programmed cell death protein 1 (PD-1) and programmed cell death protein 1 ligand (PD-L1) are the established suppressive immune checkpoints whose activation would silence the immune function [31]. Thus, immune checkpoint blockade may reactivate T lymphocytes and cancer cells are eliminated more efficiently [32]. PD-L1 blockade was taken as a representation of immunotherapy; we found that more clinical advantages and therapeutic responses to PD-L1 blockade therapy was observed in patients with high THUMPD1 expression (Figure 5B,C). Decoding the immune TME profiles of a tumor may improve the tailoring of the immunotherapeutic strategies. According to diverse functional gene expression signatures and the presence of immune-active or immunosuppressive tumor stroma and microenvironment, the TME can be subdivided into four types [24]. Targeting the selected immune checkpoint shown in Supplementary Figure S3A, we estimated the possible immunotherapeutic response in patients with different TME subtypes. After combining with the THUMPD1 expression data in TCGA, differences were observed in several cancers (Supplementary Figure S3B–M). Therein, THUMPD1 was differently expressed in four subtypes of TME in LIHC patients, suggesting diverse responses of immune checkpoint inhibitor therapy.

Through the above analysis, it may be demonstrated the direct correlation between THUMPD1 expression level and immunotherapy efficacy.

### Correlation between THUMPD1 expression and MMR defects and DNA methylation in pan-cancer

**Figure 6:**
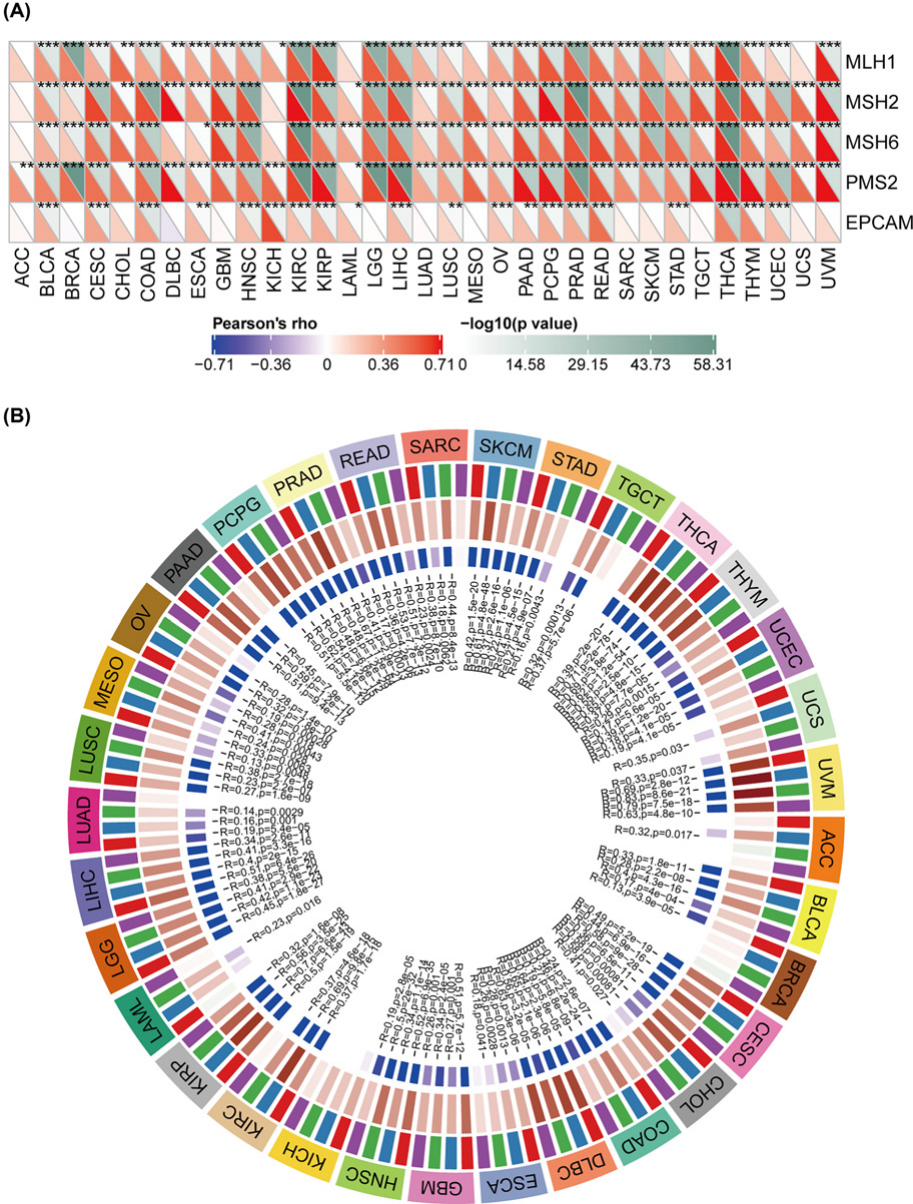
Correlation between THUMPD1 expression and MMR defects and gene methylation level in 33 cancer types from TCGA database (**A**) Correlation between THUMPD1 expression level and five MMR genes (*MLH1, MSH2, MSH6, PMS2, EPCAM*) expression. Left bottom triangle in each unit denotes coefficient of association calculated by Pearson’s correlation test, top right triangle denotes *P*-value in log10 format. (**B**) Correlation between THUMPD1 expression level and four methyltransferase genes (*DNMT1*: red, *DNMT2*: blue, *DNMT3A*: green, *DNMT3B*: purple). *P<0.05, **P<0.01, ***P<0.001.

After the exploration of the relationship between THUMPD1 expression and mutation biomarkers above, we further evaluated the association between gene expression and tumorigenesis mechanism, particularly through the MMR defects and DNA methylation of tumorigenesis essential genes. MMRs are intracellular MMR mechanism, whose functional deficiency gives rise to DNA replication errors that are unable to be repaired, causing a high level of somatic mutation. We selected five well-established MMR genes: *MLH1*, *MSH2*, *MSH6*, *PMS2* and *EPCAM*, and analyzed their mutation profiles in pan-cancer cohorts from TCGA database. According to the results, all five MMR genes were significantly correlated with THUMPD1 and the significant correlation achieved in most cancer types (Figure 6A). Interestingly, all cancer types showed a positive correlation with these MMR genes, possibly suggesting a potential role of MMR regulation in tumorigenesis. DNA methylation, as one of the DNA chemical modifications, alters genetic presentations without changing original DNA sequences. It contributes to multiple genetic alterations such as chromosomal structure, DNA stability and its interaction with certain proteins, thus regulating gene expression. We assessed the expression of four methyl transferases genes (*DNMT1,2,3,4*) in pan-cancer and investigated their correlation with THUMPD1. The results showed a co-expression with THUMPD1 in almost all cancer types (except for ACC, BRCA, kidney chromophobe (KICH) and LAML), among which the correlation coefficient was highest in THCA, UVM, PAAD, pheochromocytoma and paraganglioma (PCPG) and DLBC (Figure 6B).

### Correlation between THUMPD1 expression and gene sets from KEGG and Hallmark collections

**Figure 7:**
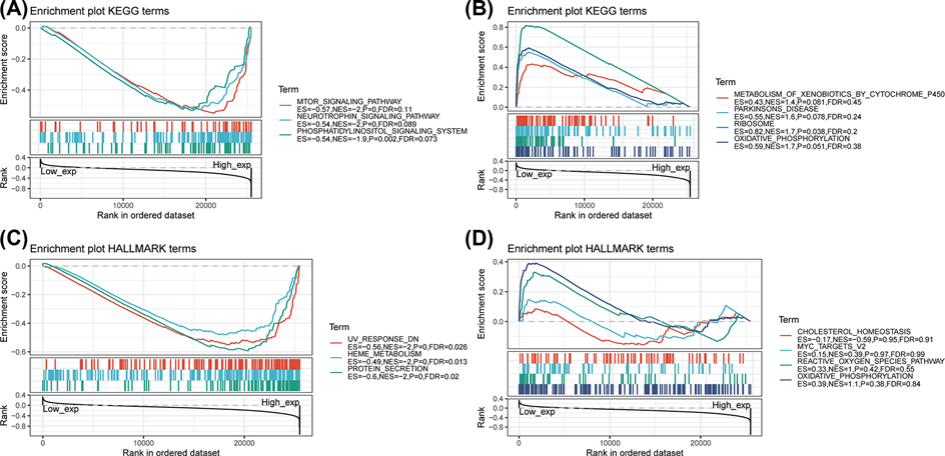
GSEA for clinical samples with low and high THUMPD1 expression (**A**) Enrichment in KEGG collection by sample with high THUMPD1 expression. (**B**) Enrichment in KEGG collection by sample with low THUMPD1 expression. (**C**) Enrichment in Hallmark term by sample with high THUMPD1 expression. (**D**) Enrichment in HALLMARK term by sample with low THUMPD1 expression. Gene sets were considered significant only when NOM *P*<0.05, FDR < 0.25.

To further explore the tumorigenic role of THUMPD1, clinical samples were divided into high- and low-expression groups with the cut-off point of the median of gene expression level. Then GSEA was conducted to investigate the pathway enrichment of THUMPD1 expression. The top three pathways with significance are shown in Figure 7. Enrichment results of KEGG collection revealed that high THUMPD1 expression was significantly correlated with mTOR signaling pathway, neurotrophin signaling pathway and phosphotidylinositol signaling system. For Hallmark term, high expression of THUMPD1 might be primarily involved in UV response, heme metabolism and protein secretion. These results gave insight into the role of THUMPD1 in cancer establishment and development.

## Discussion

THUMPD1 is a specific RNA adaptor to assist the formation of ac4C, enhancing mRNA stability and promoting its decoding efficacy [5]. It means that ac4C may affect gene expression in post-transcriptional stage, which may affect tumorigenesis-related genes expression, resulting in tumor(s) establishment. However, association between ac4C and various cancer types is still far from investigated. Hence, studies of THUMPD1 help comprehend the role of ac4C in cancer. In the present study, we performed a systemic bioinformatics analysis on THUMPD1, and investigated the differential THUMPD1 expression in pan-cancer and its correlation with various prognostic and immune regulatory indicators, based on several public databases.

According to previous studies, elevated level of urinary ac4C was discovered in patients of colorectal cancer [16], ovarian epithelial cancer [17], urogenital cancer [18] and breast cancer [19]. It can be inferred that overexpression of THUMPD1 may help the early diagnosis of certain cancer types. Here, we got THUMPD1 mRNA expression profiles of various normal and cancer tissues, which served as the basis of subsequent analyses. The results revealed that THUMPD1 expression was comparable in all 31 normal tissues, except for bone marrow and organs of urogenital systems. Additionally, gene expression largely increased in corresponding cancer tissues. Considering the interference caused by limited sample size of TCGA, we integrated data from GTEx and got comprehensive results. Most of the cancers (18/27) showed higher THUMPD1 expression than para-cancerous normal tissues, while there was also down-regulation in some cancers: BLCA, KIRC, LUSC, OV and UCEC. Besides, our results of Western blotting on KIRC and LIHC validated the expression difference among these cancer types. As ac4C is a modification of mRNA, its expression level may be affected by intensity of cellular activity. For bone marrow and cancer cells, frequent cell proliferation requires abundant mRNA to be translated into proteins for functions [33]. Therefore, high expression of THUMPD1 is required to facilitate the translation efficacy. According to our immunofluorescence results, THUMPD1 protein was diffusely distributed in the whole cell but mainly located at cytoplasm, compatible with the function in RNA binding as the cytoplasm is where RNA mainly distributes. For those cancers showing higher THUMPD1 expression, THUMPD1 can be interpreted as a novel cancer biomarker. For those cancer types with lower THUMPD1 expression, THUMPD1 may interact with cancer-specific factors or directly serve as a protective factor against cancer development. However, detailed mechanism under the differential expression results requires further investigated.

Prognostic analysis was conducted to further explore the role of THUMPD1 in diverse cancer types. To get multifaceted results, four prognostic indexes were used: OS, DSS, DFI and PFI. Our results revealed that 7 of 33 cancers showed significant HR (KIRC, LAML, LIHC, READ, THYM, CESC, PAAD), among which KIRC and READ had better prognosis consistently in OS, DSS and PFI analyses. In particular, elevated expression of THUMPD1 largely improved the survival probability of patients with READ, suggesting THUMPD1 as a potential favorable prognostic indicator for rectal cancers. Patients with LIHC and CESC, on the contrary, were subject to worse prognosis in the case of high THUMPD1 expression. These findings probably suggested THUMPD1 as an unfavorable prognostic indicator for liver and cervical cancer. Combining these results with differential mRNA expression between cancerous and adjacent normal tissues, KIRC patients had significant higher expression in normal tissue and LIHC had opposite results. Here, with distinct prognosis in high THUMPD1 expression cohorts, KIRC and LIHC were treated as the representation to support the idea that THUMPD1 may play distinct roles in different cancer types. According to pan-cancer analysis of NAT10, the partner of THUMPD1 for mRNA acetylation, high NAT10 expression is associated with worse survival in LIHC, lymphoma and colorectal cancer [34]. Although NAT10 and THUMPD1 work synergistically, their effects on prognosis of diverse cancer types are not totally the same. The underlying reasons, on the one hand, are probably that the fate of overexpressed NAT10–THUMPD1 complex is not simply an increase in ac4C level, but other unknown biological alteration that affects tumor progression as well. On the other hand, there is up- or down-regulation of specific factors that may interact with NAT10 or THUMPD1 and contribute to cancer development. Possibly, the improved or worse prognosis of patients with high THUMPD1 expression may be determined by its involvement or regulation in certain tumorigenic pathways. In our study, pathway enrichment analysis was performed to investigate the tumorigenic role of THUMPD1. GSEA results showed the enrichment of high THUMPD1 expression in mTOR signaling pathway, neurotrophin signaling pathway and phosphotidylinositol signaling system, which all stimulate aberrant cell metabolism, angiogenesis and malignant progression of tumor [35–37]. These findings, therefore, support the possible explanation that, increased THUMPD1 directly or indirectly participates and plays essential role in certain tumorigenic metabolic pathway, resulting in the different prognosis of various cancer types. Thus, THUMPD1 may be not a universal biomarker for all the cancers, but a potential predictor for prognosis of some cancer types and a valuable target worth further exploration in the mechanism of tumorigenesis.

Previous studies had demonstrated that the ac4C level was associated with inflammatory responses, which may favor subsequent cancer progression [38–40]. TILs, the immune cells inside tumor tissue as an inherent component of anti-tumor strategy, have been shown to serve as an independent predictor for cancer prognosis and efficacy of immunotherapy [25,41]. Here, we conducted a correlation analysis between THUMPD1 expression and TILs abundance in diverse cancer types. Our results revealed that the three top-ranking cancer with highest infiltration score were COAD, KIRC and LIHC, all positively correlated with THUMPD1 expression. Due to intense anti-tumor property, higher immune cell infiltration should have suggested an improved prognosis. However, LIHC and COAD patients with abundant TILs had poor prognosis in our survival analysis. Many studies had demonstrated that immune cells in TME play an essential role in cancer development and progression [42,43]. Thus, THUMPD1 may recruit and regulate TILs to promote or inhibit progression of various cancer types, indicating a crucial role in cancer immunity. In addition, the function of TILs in different cancers types may be affected by some factors, such as the ICPs and conditions of TME. In the present study, THUMPD1 expression in several cancer types, including BRCA, COAD, KIRC, and LIHC, showed significant association with most ICPs’ genes. Besides, the ICPs’ genes that exhibited significance correlation with each cancer were different. Thus, distinct prognosis for various cancers may be the consequence of up- or down-regulation of specific ICPs on the surface of immune cells. The difference in ICPs expression may be caused by THUMPD1-mediated immune cell differentiation, or by altered protein expression due to altered level of ac4C within mRNA. In addition, our analysis on TME demonstrated a close relationship between THUMPD1 expression and TMB, MSI and neoantigen in several cancer types, as well as the MMR and methyltransferase gene that showed a co-expression with THUMPD1. Previous studies have suggested a close link between TMB, MSI and response of immunotherapy, especially for immune checkpoints inhibitor, such as PD-1 inhibitor and TGF-β inhibitor [23,44–46]. Therefore, it is reasonable to speculate the THUMPD1 as an indicator for the evaluation of immune therapy efficacy for cancer patients. Based on clinical cohorts with recorded PD-L1 blockade therapy response, we discovered a significant correlation between THUMPD1 expression and immunotherapy efficacy. In most cases, cancer patients with high THUMPD1 expression are expected to respond to immunotherapy compared with those with low expression. Besides, immunotherapy response could be predicted by TME subtyping that was corresponding to different THUMPD1 expression level for some cancer types [24]. Therefore, THUMPD1 may serve as a favorable factor that predict the efficacy of immunotherapy for many cancer types. Currently, the MSI is regarded as a reliable index to assess the differentiation of COAD, and high MSI indicates better ICP inhibitor therapy response and prognosis in early and advanced clinical stages of COAD [47,48]. In our study, the TMB and MSI were both positively correlated with THUMPD1 expression in COAD patients, which supports the proposal that THUMPD1 may be a potential indicator for drug responses in several cancer types like COAD.

A general overview of the present study consistently links THUMPD1 expression to KIRC and LIHC, and suggests that THUMPD1 may be a prognostic predictor and immune therapy regulator for patients with KIRC and LIHC. The correlation between THUMPD1 expression and cancer progression and recurrence after tumor resection of KIRC and LIHC patients was consistent in different survival analyses. For KIRC, THUMPD1 expression was higher in para-cancerous normal tissue, and exhibited a better prognosis in OS, DSS and PFI survival analyses. For LIHC, on the contrary, THUMPD1 expression was lower in para-cancerous normal tissue and showed a poor prognosis. Besides, expression of THUMPD1 in KIRC and LIHC patients was both significantly correlated with immune cell infiltration, ICPs, TMB, MMRs and methyltransferase genes expression. Hence, THUMPD1 may affect tumorigenesis of KIRC and LIHC via certain pathway or mechanism, which may be suggested by enriched collections from GSEA. Additionally, besides the role in predicting cancer prognosis, THUMPD1 may also involve in regulating the immune reactions and serve as a predictor for immunotherapy efficacy. Therefore, our study is innovative to propose that THUMPD1 is a possible therapeutic target against KIRC and LIHC, expanding the multiplicity of current treatments. This proposal could also be extrapolated to other cancers showing significant correlation with THUMPD1 expression. Distinct from the results of pan-cancer analysis of NAT10, our proposition demonstrates the difference between NAT10 and THUMPD1 in clinical and immunological function, though they both serve as the writing tools of ac4C [34]. Hence, further studies are required to determine the detailed difference and the possible underlying mechanism.

Although the present study has provided evidence for the possible role of THUMPD1 in prognosis prediction and immune regulation for many cancers, it has limitations indeed. First, it is a bioinformatics analysis, so the sources of data are mostly online databases. In our study, the correlation analyses were all based on mRNA expression of THUMPD1. However, any potential modification in post-transcriptional level will lead to change of protein function and altered biological process. Even though a protein expression analysis was performed on clinical samples, more experiments are required to validate and extend our current results in future study. Second, currently demonstrated role of THUMPD1 is to assist formation of ac4C in RNA molecule, however, dynamic changes of ac4C level in tumor cells are not available. Thus, alteration of ac4C or THUMPD1 itself, the one influences cancer prognosis, immune regulation and treatment efficacy requires future investigation. Finally, the correlation between THUMPD1 expression and MMR and methyltransferase genes expression lacks reasonable interpretation from previous laboratory findings. Possibly, THUMPD1 activity may influence essential process of MMR and DNA methylation, which contribute to tumorigenesis and malignant progression in specific cancers. However, suchlike interpretation requires further experiments *in vivo* and* in vitro* to get proved. Anyway, we guide the future direction of the study on THUMPD1-mediated ac4C in clinical and transcriptome aspects.

## Conclusion

In summary, THUMPD1 is differently expressed in many cancer types, and this expression is correlated with prognosis in diverse cancers, especially for KIRC and LIHC. THUMPD1 expression also correlated with tumor infiltration of various lymphocytes, immune checkpoints, TME biomarkers, such as TMB, MSI, neoantigens, MMRs and methyltransferases. Of note, KIRC and LIHC revealed significant correlation with THUMPD1 and it is worth attention in further studies. Taken together, our study proposed that THUMPD1 may be a novel predictor to evaluate the prognosis and immune therapy efficacy in diverse cancer types.

## Supplementary Material

Supplementary Figures S1-S3

## Data Availability

Our study took advantage of public sources. The data can be found in: CCLE: https://sites.broadinstitute.org/ccle; UCSC Xena platform: http://xena.ucsc.edu; TIMER: https://cistrome.shinyapps.io/timer/

## Abbreviations

ACC: adrenocortical carcinoma
ac4C: N4-acetylcytidine
BLCA: bladder urothelial carcinoma
BRCA: breast invasive carcinoma
CCLE: Cancer Cell Line Encyclopedia
CESC: cervical and endocervical cancer
CI: confidence interval
COAD: colon adenocarcinoma
DAPI: 4',6-diamidino-2-phenylindole
DFI: disease-free interval
DLBC: diffuse large B-cell lymphoma
DSS: disease-specific survival
FDR: false discovery rate
GSEA: gene set enrichment analysis
GTEx: Genotype-Tissue Expression
HNSC: head and neck squamous cell carcinoma
HR: hazard ratio
KEGG: Kyoto Encyclopedia of Genes and Genomes
KICH: kidney chromophobe
KIRC: kidney renal clear cell carcinoma
LAML: acute myeloid leukemia
LIHC: liver hepatocellular carcinoma
LUAD: lung adenocarcinoma
LUSC: lung squamous cell carcinoma
MMR: mismatch repair
MSI: microsatellite instability
NAT10: N-acetyltransferase 10
OS: overall survival
OV: ovarian serous cystadenocarcinoma
PAAD: pancreatic adenocarcinoma
PBS: phosphate-buffered saline
PD-1: programmed cell death protein 1
PD-L1: programmed cell death protein 1 ligand
PFI: progression-free interval
PRAD: prostate adenocarcinoma
READ: rectum adenocarcinoma
SKCM: skin cutaneous melanoma
STAD: stomach adenocarcinoma
TCGA: The Cancer Genome Atlas
THCA: thyroid carcinoma
THUMPD1: THUMP domain-containing protein 1
THYM: thymoma
TIL: tumor-infiltrating lymphocyte
TIMER: Tumor Immune Estimation Resource
TME: tumor microenvironment
TMB: tumor mutational burden
UCEC: uterine corpus endometrial carcinoma
UCS: uterine carcinosarcoma
UVM: uveal melanoma
