# Supplementary material for: Pan-cancer analysis of N4-acetylcytidine adaptor THUMPD1 as a predictor for prognosis and immunotherapy
Source: Biosci Rep. 2021 Dec 7;41(12):BSR20212300. doi: 10.1042/BSR20212300 (PMC8655504; doi:10.1042/BSR20212300)
Supplement: Supplementary Figures S1-S3 [file BSR-2021-2300_supp.pdf]

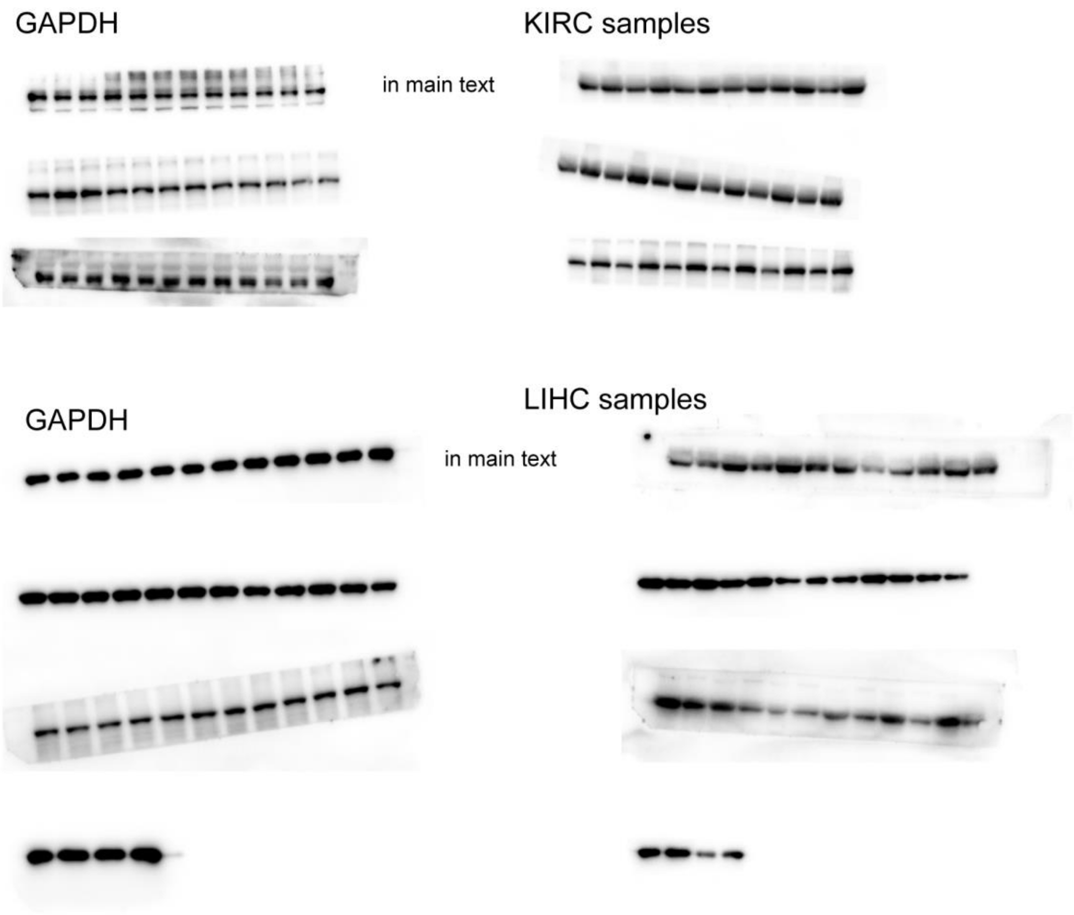


**Supplementary Figure 1**. Original bands of Western blots in KIRC and LIHC clinical samples.

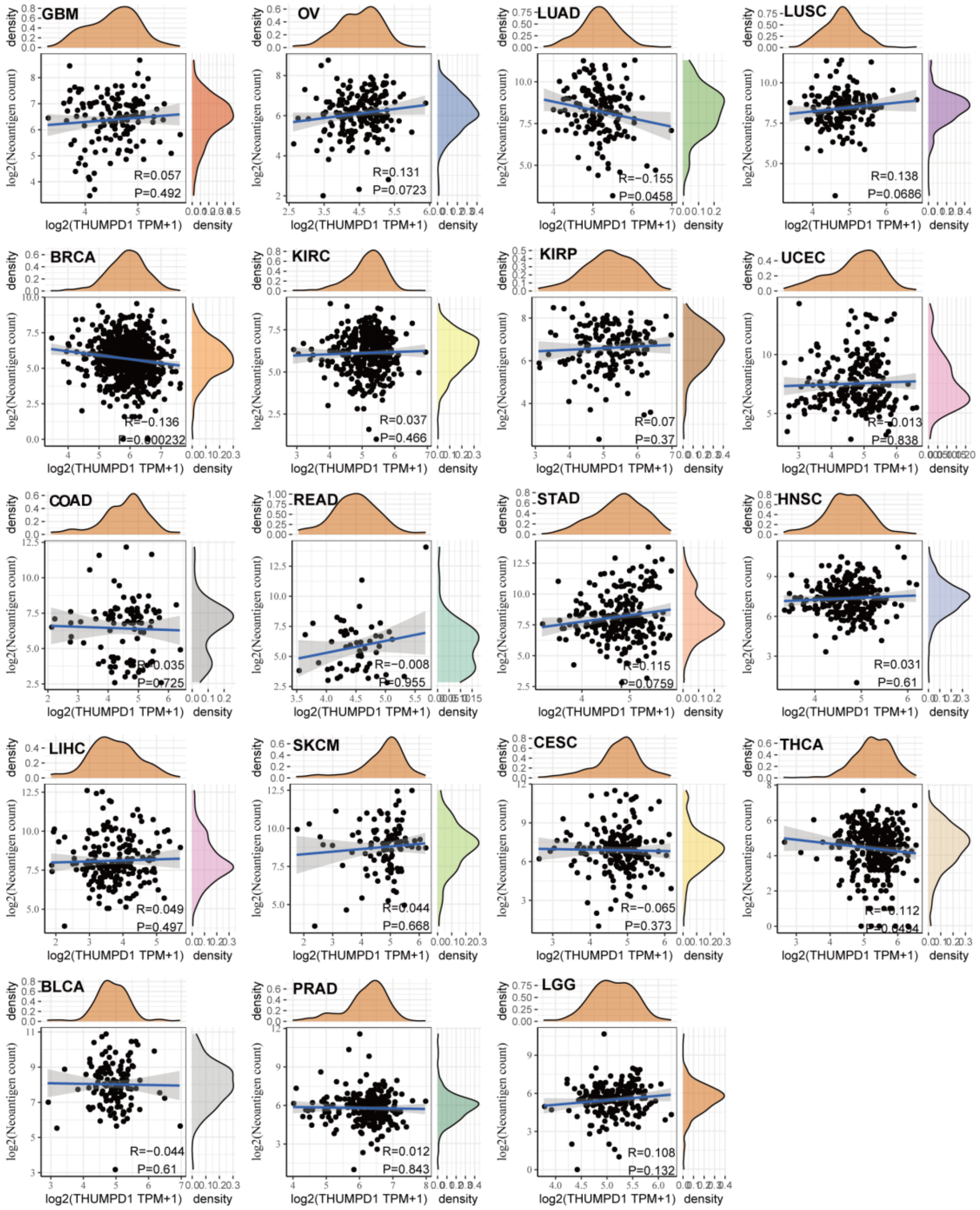


**Supplementary Figure 2.** Correlation between THUMPD1 expression and neoantigen in 33 cancer types from TCGA database.

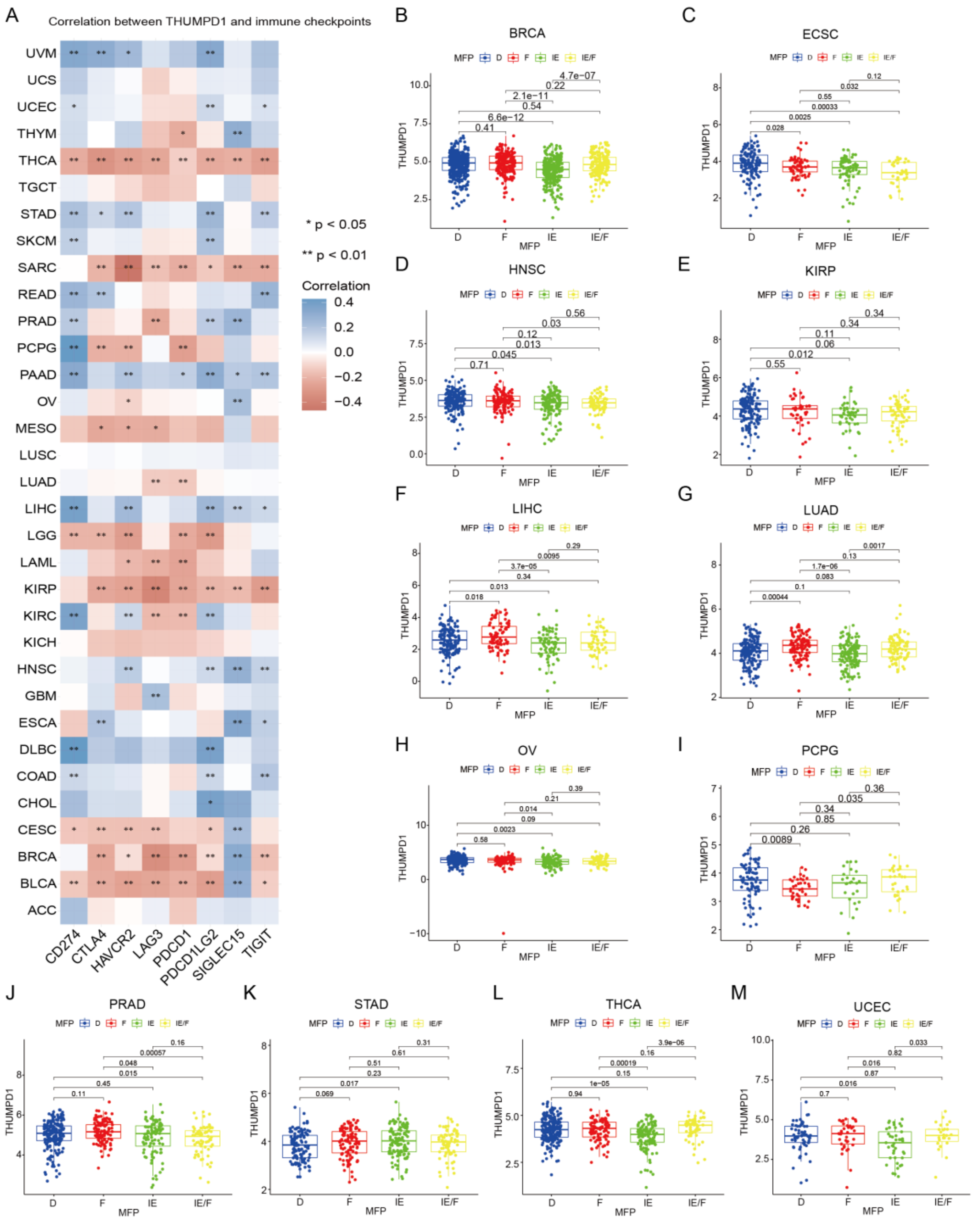


**Supplementary Figure 3. Immunotherapy response prediction.**

(**A**) Heat map describing the correlation between THUMPD1 expression and selected immune checkpoint genes. (**B-M**) Correlation between THUMPD1 expression and TME subtypes in BRCA, ECSC, HNSC, KIRP, LIHC, LUAD, OV, PCPG, PRAD, STAD, THCA and UCEC. D: immune-depleted; F: fibrotic; IE: immune-enriched; IE/F: immune-enriched, fibrotic. MFP: molecular function portrait.
